# Transcending technique: a theoretical framework and empirical study of sports dance appreciation behavior among university students in Guangzhou

**DOI:** 10.3389/fpsyg.2026.1750644

**Published:** 2026-02-26

**Authors:** Yuanmei He

**Affiliations:** School of Physical Education, Guangzhou University, Guangzhou, China

**Keywords:** aesthetic psychology, appreciation behavior, art reception theory, cluster analysis, principal component analysis, sports dance, university students

## Abstract

This study employs empirical methods to investigate the essence of sports dance appreciation behavior among university students in the Guangzhou area. An online questionnaire survey was distributed to 350 students from universities across Guangzhou, including comprehensive and technological institutions. Utilizing Principal Component Analysis (PCA) and cluster analysis, this research identified three core dimensions of sports dance appreciation: Intuitive-Emotional, Technical-Analytical, and Formal-Aesthetic. Furthermore, three distinct typologies of appreciators were established: Emotional Resonators (45.1%), Technical Evaluators (30.0%), and Comprehensive Appreciators (24.9%). The findings reveal that the sports dance appreciation behavior of Guangzhou university students exhibits significant subjectivity and diversity, with their appreciation preferences being substantially influenced by regional cultural background and personal experience. The developed Sports Dance Appreciation Experience Scale demonstrated excellent psychometric properties, with PCA revealing a three-factor structure accounting for 56.15% of the total variance. Significant associations were found between appreciation types and demographic variables such as gender, academic major, and prior dance training, highlighting the roles of cognitive style and embodied experience in shaping aesthetic processing. Drawing on art reception theory, aesthetic psychology, and aesthetic education pedagogy, this study constructs a theoretical framework for sports dance appreciation and provides empirical evidence for understanding the aesthetic reception mechanisms of this hybrid art-sport form. These insights offer important implications for designing differentiated aesthetic education strategies and curriculum development in sports dance.

## Introduction

Sports dance, as a hybrid genre, occupies a unique and dynamic space within the broader spectrum of aesthetic and physical practices (Bai, 2005; Best, 1974). It represents a fascinating synthesis of disciplined athleticism and expressive artistry, demanding not only technical precision and physical endurance but also a capacity for emotional storytelling and aesthetic communication. This fusion creates a complex perceptual object for audiences, who must navigate its dual nature as both a competitive sport and a performing art.

In Guangzhou’s higher education institutions, sports dance has gained substantial popularity, serving not only as a physical education activity but also as an important medium for cultural exchange and aesthetic education (Boiché and Stephan, 2014). The city’s distinctive position as the cultural center of the Lingnan region, with its rich heritage of Cantonese opera, traditional folk arts, and a history of cosmopolitan exchange, creates a particularly interesting and fertile context for examining how contemporary university students engage with and appreciate this global yet locally interpreted art form (Bourdieu, 1984).

The academic study of sports dance has traditionally emphasized technical execution and competitive performance standards, with substantial literature focusing on biomechanical analysis, training methodologies, and judging criteria (Cerasoli et al., 2014; Chatterjee and Vartanian, 2014). This production-oriented paradigm has yielded valuable insights into the objective determinants of performance quality. However, this predominant focus on the productive aspects of sports dance has created a significant gap in understanding the receptive dimension - how audiences, particularly student populations, perceive, process, and derive meaning from sports dance performances (Chen et al., 1999).

This gap becomes especially pertinent given the growing emphasis on aesthetic education in Chinese higher education policy and the recognized importance of cultivating comprehensive aesthetic literacy among university students (Chen, 2020). Understanding how students appreciate sports dance is crucial for designing effective pedagogical interventions that can enhance their aesthetic sensitivity and cultural understanding.

The theoretical foundation of this research integrates three complementary perspectives to address this gap. First, art reception theory, particularly as developed in the works of Jauss (Corpus and Wormington, 2014) and Iser (Cross, 2025), illuminates how audiences are not passive recipients but active co-creators of meaning through their engagement with artistic works. Their “horizon of expectations,” shaped by prior cultural and personal experiences, fundamentally influences their interpretation and appreciation. Second, aesthetic psychology, from the early work of Fechner to contemporary models like Leder’s (Csikszentmihalyi, 1990), examines the cognitive and emotional processes underlying aesthetic experiences, including perception, classification, memory integration, and emotional evaluation.

Third, aesthetic education pedagogy, drawing from philosophers like Schiller (Dewey, 1934) and Dewey (Doosje et al., 1995), provides frameworks for systematically developing aesthetic appreciation capabilities, emphasizing the cultivation of perception, critical reflection, and emotional responsiveness.

Recent developments in neuroaesthetics have further enriched our understanding of aesthetic experiences by revealing their biological underpinnings (Gadamer, 1960; Iser, 1978). Studies utilizing functional magnetic resonance imaging (fMRI) and electroencephalography (EEG) have demonstrated that aesthetic appreciation involves distributed neural networks spanning sensory, emotional, valuation, and cognitive domains (Jauss, 1982; Kant, 1790). For instance, the perception of beauty correlates with activity in the medial orbitofrontal cortex, while the experience of awe or being “moved” involves the amygdala and insula. The mirror neuron system is also implicated, suggesting a neural basis for the embodied understanding of observed movement (Leder et al., 2004; Nadal and Skov, 2018).

The current study, therefore, addresses several critical and interconnected research gaps. First, it decisively shifts the academic focus from the technical production of sports dance to its reception and appreciation by a specific, culturally situated audience—university students. Second, it empirically examines how the unique regional cultural context of Guangzhou, with its Lingnan heritage, influences aesthetic preferences and appreciation patterns, exploring the concept of “horizon fusion” (Orgs et al., 2013). Third, it moves beyond monolithic views of “the audience” to develop a comprehensive, empirically derived typology of sports dance appreciators, which can inform differentiated educational approaches. Finally, it integrates theoretical perspectives from art reception, psychology, neuroscience, and education to create a multidimensional and robust understanding of sports dance appreciation behavior.

## Materials and methods

### Research design and participants

This study employed a Convergent Mixed-Methods Design (QUAN + qual), where quantitative and qualitative data were collected concurrently. While data collection was simultaneous, the data analysis followed a sequential explanatory logic: the quantitative typologies were established first, which then guided the directed content analysis of the qualitative responses to provide context and depth to these specific types. The research was conducted over a six-month period from March to August 2023.

A stratified random sampling method was used to select 350 undergraduate students from five universities located in the Guangzhou Higher Education Mega Center. The sample comprised a comprehensive research university, a leading technological institution, a university with a strong humanities focus, a teacher-training university, and a municipal comprehensive university. These institutions were strategically selected to represent the diversity of higher education in Guangzhou. The sample size was determined through *a priori* power analysis using G*Power software for a multiple regression analysis. Following Cohen’s (1988) conventional benchmarks for social science research, we adopted a medium effect size (f^2^ = 0.15) to ensure a conservative estimation of statistical power, with an alpha error probability of 0.05, and a desired statistical power of 0.95. The demographic characteristics of the sample are presented in Table 1.

**Table 1:** Sample demographic distribution (*N* = 350).

Demographic variable	Category	Number	Percentage (%)
Gender	Male	170	48.6
	Female	180	51.4
Academic year	Freshman	95	27.1
	Sophomore	105	30.0
	Junior	88	25.1
	Senior	62	17.7
Major	Humanities/Social sciences	126	36.0
	Science/Engineering	159	45.4
	Arts	65	18.6
Dance training experience	Yes	58	16.6
	No	292	83.4
Monthly viewing frequency	Once or less	198	56.6
	2–3 times	112	32.0
	4 times or more	40	11.4

### Instrument development and validation

The research instrument was a carefully constructed questionnaire consisting of three main components: a demographic information section, the Sports Dance Appreciation Experience Scale (SDAES), and a section with open-ended qualitative questions. The development of the SDAES followed a rigorous, multi-stage process adhering to established psychometric principles to ensure its content validity, construct validity, and reliability.

In the initial stage, a pool of 40 items was generated through a comprehensive and iterative process. This involved a systematic literature review of theoretical frameworks from art reception theory, aesthetic psychology, and sports esthetics to identify key constructs. Expert consultations were then conducted with five distinguished professors specializing in sports science, dance education, and aesthetic psychology. These experts evaluated the initial item pool for content validity, theoretical relevance, clarity, and comprehensiveness, providing critical feedback for refinement. Subsequently, focus group discussions were held with 20 students from different academic backgrounds (Humanities, STEM, Arts) who were not part of the main study.

The initial 40-item pool was then subjected to a formal content validity assessment using the Content Validity Index (CVI). Three independent experts rated each item on a 4-point scale for relevance, clarity, and comprehensiveness. Items with a CVI score below the recommended threshold of 0.78 were eliminated or substantially revised, resulting in a refined 30-item preliminary scale. This preliminary scale was then administered to a pilot sample of 50 students from a university not involved in the main study, the pilot data were analyzed using Principal Component Analysis (PCA) with Varimax rotation. PCA was selected primarily for data reduction purposes at this initial stage to identify the most salient components explaining the maximum variance and to examine the underlying factor structure and reliability analysis was conducted to assess internal consistency. Items with low factor loadings (<0.50) or those that cross-loaded on multiple factors were removed to purify the scale.

The final Sports Dance Appreciation Experience Scale comprised 23 items distributed across three clear dimensions, using a standard 5-point Likert scale ranging from 1 (strongly disagree) to 5 (strongly agree). The reliability analysis results are shown in Table 2.

**Table 2:** Questionnaire reliability analysis results.

Dimension	Number of items	Cronbach’s α	Split-half reliability
Intuitive-Emotional	8	0.896	0.894
Technical-Analytical	7	0.894	0.898
Formal-Aesthetic	8	0.847	0.831

### Data collection procedure

The study was reviewed and approved by the Ethics Committee of Guangzhou University (Approval No. 2023-66). We Nonetheless, we strictly adhered to research ethics by providing a detailed information sheet at the beginning of the online survey and obtaining explicit consent (via a checked box) from all participants. All data were analyzed and reported in aggregate form.

Data collection was conducted entirely online via the Qualtrics platform. A link to the questionnaire was distributed to potential participants through university student portals, official course groups, or campus email lists. This approach ensured broad and efficient reach across the five participating universities while maintaining a standardized administration procedure.

The online questionnaire was designed to be concise, and the average completion time, as recorded by the survey platform, was approximately 10 min. To minimize potential order effects, the presentation sequence of the scale items was randomized for half of the participants. The qualitative component consisted of three strategically designed open-ended questions placed at the end of the questionnaire to avoid influencing the quantitative responses.

### Data analysis strategy

The data analysis employed a comprehensive and integrated mixed-methods approach, where quantitative and qualitative analyses were conducted concurrently and then merged during the interpretation phase to provide a holistic understanding. All quantitative analyses were performed using SPSS and AMOS software, while qualitative data were managed and analyzed using NVivo.

The quantitative analysis proceeded through several sequential and interconnected stages. First, descriptive statistics were computed for all demographic variables and scale items. Second, Principal Component Analysis (PCA) was conducted on the 23-item scale. Third, to validate the factor structure identified through PCA, Confirmatory Factor Analysis (CFA) was performed using maximum likelihood estimation. Fourth, the internal consistency reliability of the overall scale and each sub-dimension was assessed using Cronbach’s alpha coefficients. Fifth, to identify distinct typologies of appreciators, a K-means clustering analysis was conducted using the standardized factor scores of the three appreciation dimensions as input variables. Finally, Chi-square tests and Analysis of Variance (ANOVA) were used to examine the relationships between cluster membership and demographic variables.

The qualitative analysis followed a systematic thematic content analysis procedure. First, all responses to the open-ended questions were transcribed verbatim into digital text and imported into NVivo. Second, an initial coding framework was developed deductively based on key theoretical concepts from the literature and inductively from emergent themes that arose directly from the data itself. Third, to ensure coding reliability, two researchers independently coded a randomly selected subset of 50 responses. Inter-coder reliability was calculated, yielding a Cohen’s kappa coefficient of 0.87, indicating a high level of agreement.

## Results

### Psychometric properties of the sports dance appreciation scale

The Principal Component Analysis revealed a robust and theoretically coherent three-factor structure. The KMO measure was 0.941, and Bartlett’s test was significant (χ^2^ = 3632.73, *p* < 0.001). The three factors collectively accounted for 56.15% of the total variance in the 23-item appreciation scale. The Intuitive-Emotional dimension explained 20.62% of the variance, followed by the Technical-Analytical dimension (18.81%), and the Formal-Aesthetic dimension (16.72%). All items exhibited significant factor loadings ranging from 0.56 to 0.77 on their primary factors with minimal cross-loadings. The detailed PCA results are presented in Table 3.

**Table 3:** Component Loadings for Sports Dance Appreciation Scale (Selected Marker Items)

**Item Code**	**Item Description**	**C1**	**C2**	**C3**	**Comm.**
**Technical**	**(Technical-Analytical Component)**				
TA_7	I compare the technical skills of different dancers	**.768**	-	-	.668
TA_3	I analyze if the partnership is harmonious	**.736**	-	-	.622
TA_5	I evaluate the difficulty and execution quality	**.727**	-	-	.594
TA_6	I focus on rhythm and coordination with music	**.714**	-	-	.597
**Formal**	**(Formal-Aesthetic Component)**				
FA_7	I value the overall harmony and balance	-	**.728**	-	.545
FA_6	Color coordination affects my experience	-	**.698**	-	.499
FA_2	I focus on stage setting and lighting effects	-	**.696**	-	.518
FA_8	Design of beginning and ending influences me	-	**.681**	-	.524
**Intuitive**	**(Intuitive-Emotional Component)**				
IE_5	Rhythm and dynamism touch my heart	-	-	**.750**	.581
IE_8	Performance evokes physical reactions	-	-	**.737**	.614
IE_3	Emotional expression deeply affects me	-	-	**.734**	.594
IE_4	I experience strong emotional resonance	-	-	**.730**	.602
	**Eigenvalue**	7.50	4.18	1.23	-
	**Variance Explained (%)**	18.81	16.72	20.62	-
	**Cumulative Variance (%)**	18.81	35.53	**56.15**	-

Confirmatory Factor Analysis provided strong additional support for the three-factor model identified through PCA. The model fit indices were all excellent: the normed chi-square (χ^2^/df) was 2.34 (*p* < 0.001), the Comparative Fit Index (CFI) was 0.941 and the Tucker-Lewis Index (TLI) was 0.928. The Root Mean Square Error of Approximation (RMSEA) was 0.062 with a 90% confidence interval of 0.055–0.069. The Standardized Root Mean Square Residual (SRMR) was 0.043. All standardized factor loadings in the CFA were statistically significant (*p* < 0.001) and exceeded 0.60, providing strong evidence for convergent validity.

### Appreciator typology through cluster analysis

The K-means cluster analysis was conducted based on the factor scores of the three appreciation dimensions. Prior to clustering, raw scores were standardized using Z-scores to prevent scale differences from biasing the results. The determination of the three-cluster solution was guided by a combination of statistical criteria (visual inspection of the dendrogram and agglomeration coefficients) and theoretical interpretability, revealing three distinct and meaningful appreciator types A one-way ANOVA confirmed that there were significant differences across all three appreciation dimensions between the clusters (*p* < 0.001 for all dimensions). The clustering solution demonstrated good stability and quality, with silhouette coefficients ranging from 0.71 to 0.84.

The three clusters were characterized and labeled based on their distinct score profiles (Table 4):

1. Emotional Resonators (*n* = 158, 45.1%): This largest cluster was defined by very high scores on the Intuitive-Emotional dimension but markedly lower scores on the Technical-Analytical and Formal-Aesthetic dimensions.
2. Technical Evaluators (*n* = 105, 30.0%): This group demonstrated the highest score on the Technical-Analytical dimension, with moderate scores on the Formal-Aesthetic dimension and the lowest score on the Intuitive-Emotional dimension.
3. Comprehensive Appreciators (*n* = 87, 24.9%): This smallest cluster exhibited balanced and high scores across all three dimensions, with particularly strong performance on the Formal-Aesthetic dimension.

**Table 4:** Distribution of cluster centers for sports dance appreciator types.

Appreciation dimension	Emotional resonators (*n* = 158)	Technical evaluators (*n* = 105)	Comprehensive appreciators (*n* = 87)	*F*-value	*p*-value
Intuitive-Emotional	4.52 ± 0.43	3.12 ± 0.56	4.23 ± 0.38	98.76	<0.001
Technical-Analytical	2.89 ± 0.61	4.45 ± 0.42	4.12 ± 0.47	127.43	<0.001
Formal-Aesthetic	3.24 ± 0.52	3.56 ± 0.48	4.38 ± 0.41	89.54	<0.001

A follow-up discriminant analysis was conducted to validate the cluster solution. The analysis correctly classified 90.6% of the original grouped cases, providing strong external validation for the three-cluster typology. Wilks’ lambda was 0.136 (*p* < 0.001), indicating that the group means are significantly different.

### Demographic variations in appreciation patterns

Significant associations were found between appreciator types and key demographic characteristics (Table 5). Gender differences were particularly notable. Males were significantly overrepresented in the Technical Evaluator cluster (59.0% of Technical Evaluators were male, compared to 41.0% female, *p* < 0.01). Conversely, females showed a higher tendency toward being Emotional Resonators (57.0%) and Comprehensive Appreciators (54.0%).

**Table 5:** Comparison of demographic characteristics by appreciator type.

Demographic variable	Emotional resonators (*n* = 158)	Technical evaluators (*n* = 105)	Comprehensive appreciators (*n* = 87)	χ^2^	*p*-value
Gender*				6.78	0.004
Male	68 (43.0%)	62 (59.0%)	40 (46.0%)		
Female	90 (57.0%)	43 (41.0%)	47 (54.0%)		
Major**				12.24	<0.001
Humanities/Soc Sci	65 (41.1%)	32 (30.5%)	29 (33.3%)		
Science/Engineering	68 (43.0%)	58 (55.2%)	33 (37.9%)		
Arts	25 (15.8%)	15 (14.3%)	25 (28.7%)		
Dance experience***				13.49	<0.001
Yes	15 (9.5%)	28 (26.7%)	15 (17.2%)		
No	143 (90.5%)	77 (73.3%)	72 (82.8%)		

*p < 0.01, **p < 0.001, ***p < 0.001.

Academic major also showed a strong and theoretically coherent association with appreciation patterns. Science and Engineering students comprised 55.2% of Technical Evaluators but only 37.9% of Comprehensive Appreciators. Conversely, Arts majors were substantially overrepresented in the Comprehensive Appreciator cluster (28.7% vs. 14.3–15.8% in other clusters).

Students from Humanities and Social Sciences were most prevalent among Emotional Resonators (41.1%).

Dance training experience showed the expected pattern, with students who had formal training being significantly more likely to be classified as Technical Evaluators (26.7%) or Comprehensive Appreciators (17.2%) rather than Emotional Resonators (9.5%).

### Qualitative characterization of appreciator types

The qualitative thematic analysis provided rich, nuanced descriptions that vividly brought to life the three appreciator types identified quantitatively, revealing distinct patterns in how they experience, describe, and evaluate sports dance (Table 6).

**Table 6:** Qualitative feature analysis of different appreciator types.

Appreciator type	Core characteristics	Typical statement examples	Representative keywords
Emotional Resonators	Focus on intuitive experience, emotional connection	“The passion of Latin dance reminds me of the vitality of Guangzhou summer nights; you do not need to understand the technique to be moved by it.”	Moved, infected, intuition, atmosphere
Technical Evaluators	Focus on technical standards, movement details	“I pay attention to whether the dancers’ footwork is standard and if the partnership is synchronized.”	Standard, details, technique, norms
Comprehensive Appreciators	Balance technique, emotion, and form	“An excellent performance requires the perfect integration of technique, emotion, and staging; none can be missing.”	Holistic, coordinated, perfect, balance

*Emotional Resonators* consistently emphasized immediate, intuitive experiences and deep emotional connections. Their descriptions were often metaphorical, impressionistic, and highly personal. For example, one student wrote, “The passion of Latin dance reminds me of the vitality of Guangzhou summer nights; you do not need to understand the technique to be moved by it.”

*Technical Evaluators* employed a more systematic, detached, and criteria-based approach to appreciation. Their focus was squarely on observable technical elements and the execution of movement according to perceived standards. Their responses frequently included comparative assessments and demonstrated substantial knowledge of dance terminology. A representative statement was: “I pay attention to whether the dancers’ footwork is standard and if the partnership is synchronized. I notice if their frame is maintained during turns and if their timing is precise with the music.”

*Comprehensive Appreciators* demonstrated a sophisticated, integrated approach that consciously balanced emotional engagement with technical understanding and formal analysis. They exhibited a clear awareness of the interconnectedness of different performance elements and often situated their appreciation within broader artistic or cultural contexts. One student articulated this holistic view: “An excellent performance requires the perfect integration of technique, emotion, and staging; none can be missing. The technique serves the emotion, and the staging enhances it all, creating a unified story.”

## Discussion

### Theoretical integration of appreciation dimensions

The clear three-dimensional structure of sports dance appreciation identified in this study provides robust empirical support for integrated theoretical models of aesthetic experience that span affective, cognitive, and perceptual domains (Csikszentmihalyi, 1990; Jauss, 1982). The Intuitive-Emotional dimension corresponds closely with what neuroaesthetics researchers have termed the “emotional route” or “aesthetic empathy” pathway to aesthetic appreciation (Kant, 1790; Orlandi et al., 2024). This route theoretically aligns with subcortical limbic system structures, including the amygdala (for salience and arousal) and the insula (for visceral and emotional feelings), suggesting an association between these regions and the intuitive processing observed in our participants.

The Technical-Analytical dimension aligns squarely with the cognitive-evaluative aspects of aesthetic experience, engaging prefrontal cortical regions associated with executive function, working memory, rule application, and comparative judgment (Gadamer, 1960). The strong emphasis on technical standards, details, and the importance of background knowledge in this dimension reflects what philosophers like Kant have described as “dependent beauty” (Pelowski et al., 2017) – appreciation that relies on understanding an object’s purpose, function, and its perfection relative to its type or category.

The Formal-Aesthetic dimension represents an interesting integration of mid-level sensory processing and higher-order cognitive integration. It likely involves interactions between occipital and temporal visual processing regions (for analyzing shapes, patterns, and composition) and structures within the dPCAult mode network (DMN), such as the medial prefrontal cortex and posterior cingulate cortex, which are involved in self-referential thought, contextual integration, and narrative construction (Ryan et al., 2008). This dimension’s focus on compositional elements, harmony, and holistic integration resonates with classical Gestalt theories of perception and more recent theories of “aesthetic perception” that emphasize the active detection of formal relations, unity, and complexity (Schiller, 1794).

### Demographic influences and their implications

The significant demographic variations in appreciation patterns reveal how deeply individual differences and socialization processes shape aesthetic engagement. The pronounced gender difference, with males significantly overrepresented in the Technical Evaluator cluster, aligns with a substantial body of psychological research on cognitive styles (Sorenson and Caprio, 1998). This research often suggests a greater male propensity, on average, for “systemizing” - the drive to analyze, understand, and construct rule-based systems.

The strong association between academic major and appreciation type powerfully underscores how disciplinary training cultivates distinct perceptual habits and evaluative frameworks (Tschacher et al., 2012). Science and Engineering students’ pronounced tendency toward technical evaluation reflects the analytical, criteria-based, and problem-solving thinking rigorously cultivated in STEM fields. Conversely, Arts students balanced, comprehensive approach demonstrates the interdisciplinary perspective and the integration of theory, practice, and critique fostered in arts education.

The role of dance training experience highlights the fundamental importance of embodied knowledge in aesthetic appreciation (Leder et al., 2004; Wann et al., 2001). The finding that students with formal technical training were more likely to be Technical Evaluators or Comprehensive Appreciators strongly supports theories of “embodied cognition” and “simulation theory,” which posit close links between motor experience and perceptual understanding. Observing a complex movement sequence is facilitated by the observer’s own motor repertoire for that action; they can, in a sense, “feel” the movement in their own bodies.

### Cultural context and horizon fusion

The qualitative findings vividly reveal how Guangzhou students’ appreciation of sports dance reflects a dynamic and creative process of “horizon fusion” (Horizontverschmelzung), a concept from Gadamer’s hermeneutics (Orgs et al., 2013), between traditional Lingnan cultural frameworks and globalized dance forms. The frequent, often subconscious, references to Cantonese opera rhythms, the use of local cultural metaphors (e.g., comparing the fluidity of a waltz to the Pearl River), and the valuation of communal emotional expression demonstrate how students actively integrate new aesthetic experiences within their existing cultural schemas (Bourdieu, 1984).

The emerging neuroscience of cultural cognition provides a potential mechanistic explanation for this horizon fusion (Warburton, 2011). It suggests that culturally acquired semantic networks and value systems physically shape how sensory information is processed and interpreted in the brain. When a Guangzhou student perceives an implicit similarity between the rhythmic structure of a Samba and the percussion patterns of Cantonese opera, they are likely activating overlapping neural networks that link the auditory cortex with culturally specific associative regions in the temporal and frontal lobes.

### Educational applications and curriculum design

The empirically identified appreciation typology provides a valuable, student-centered framework for differentiated aesthetic education strategies (Chen, 2020; Watson et al., 1988). Moving beyond a monolithic view of the student audience allows for targeted pedagogical interventions.

For *Emotional Resonators*, who possess a strong intuitive connection but lack analytical language, educational approaches should aim to gently develop technical vocabulary and analytical frameworks without sacrificing their innate strength in emotional engagement (Xunzi, 1979; Zeng, 2000). This might involve “guided observation” exercises where they are asked to pinpoint which specific moment in a performance elicited a strong feeling and then analyze the technical or formal elements that contributed to that emotional impact.

*Technical Evaluators* would benefit most from educational experiences that deliberately broaden their appreciation beyond a narrow focus on technical proficiency to include the emotional and formal dimensions. For Technical Evaluators, we propose interventions such as an “Emotion Identification Workshop,” explicitly tasking them to link specific technical movements to affective descriptors, thereby broadening their evaluative scope. Comparative analysis of different professional interpretations of the same dance piece could help these students recognize the validity and impact of varied artistic choices beyond mere technical correctness.

*Comprehensive Appreciators* represent the ideal outcome of aesthetic education, demonstrating a mature, integrated engagement. Educational strategies for this group should focus on deepening their already sophisticated understanding and critical perspective. This could involve research projects exploring the historical or sociocultural context of a particular dance style, critical reviews that require them to synthesize all three dimensions of appreciation.

## Conclusion

This study establishes and validates a comprehensive three-dimensional model (Intuitive-Emotional, Technical-Analytical, Formal-Aesthetic) that integrates the affective, cognitive, and perceptual aspects of aesthetic experience into a single framework for understanding sports dance appreciation. It identifies a robust typology of three distinct appreciator types (Emotional Resonators, Technical Evaluators, Comprehensive Appreciators) and empirically demonstrates how specific demographic factors and cultural context profoundly shape these appreciation patterns.

The findings provide strong empirical support for theoretical concepts from art reception theory, particularly the notion of “horizon fusion” in cross-cultural aesthetic encounters, and ground psychological models of appreciation in the specific context of a hybrid art-sport. The research extends existing aesthetic theories by rigorously applying them to the understudied domain of sports dance, revealing both commonalities with the appreciation of traditional art forms and unique characteristics stemming from its hybrid nature.

Based on the research findings, we recommend the adoption of a differentiated instructional strategy in sports dance education that acknowledges and addresses the diverse entry points and strengths of students. Sports dance appreciation courses should explicitly structure learning objectives, teaching materials, and assessment methods around all three appreciation dimensions and their synthesis. The integration of practical movement experiences appears particularly promising for developing the embodied understanding that bridges different appreciation dimensions.

### Limitations and future research directions

While this study provides valuable insights, several limitations should be acknowledged. First, the sample was geographically limited to universities in Guangzhou. This cultural specificity is a strength in depth but may limit the generalizability of the findings to other regions of China or other national contexts with different cultural and educational traditions. Future comparative research should examine whether similar appreciation dimensions and typologies emerge in different regional and national settings. Additionally, the observed “horizon fusion” is likely influenced by the specific Lingnan cultural heritage of Guangzhou. Therefore, the typologies identified here are situated within this specific cultural context and may not be universally generalizable without further comparative research.

Second, the cross-sectional design provides a detailed snapshot of appreciation patterns at a single point in time but cannot track their development or plasticity. Longitudinal studies following students through a semester- or year-long aesthetic education program could reveal how appreciation capacities evolve naturally and what specific educational approaches most effectively promote development toward a more comprehensive appreciation style.

Third, the reliance on self-report measures, while methodologically appropriate and necessary for capturing subjective experiences, could be powerfully complemented by multimodal physiological and behavioral measures in future research. Eye-tracking technology could reveal distinct visual attention patterns associated with different appreciation styles. EEG or fMRI could be used to identify the neural correlates of the three appreciation dimensions, providing a biological validation of the model.

Finally, future research should move beyond description to intervention. Experimental studies comparing the effectiveness of different pedagogical approaches tailored to specific appreciation types could provide concrete evidence for the practical utility of the typology developed in this study and help refine the recommended educational strategies. It is important to note that our use of Principal Component Analysis (PCA) was intended primarily for initial data reduction. While appropriate for this stage, future validation studies may employ Principal Axis Factoring (PAF) or Confirmatory Factor Analysis (CFA) on new samples to further verify the stability of the latent structure.

## Data Availability

The original contributions presented in the study are included in the article/Supplementary material, further inquiries can be directed to the corresponding author.
